# Helicases in R-loop Formation and Resolution

**DOI:** 10.1016/j.jbc.2023.105307

**Published:** 2023-09-29

**Authors:** Shizhuo Yang, Lacey Winstone, Sohaumn Mondal, Yuliang Wu

**Affiliations:** Department of Biochemistry, Microbiology and Immunology, University of Saskatchewan, Saskatoon, Saskatchewan, Canada

**Keywords:** R-loop, helicase, RNA-DNA hybrid, R-loop formation, R-loop resolution, CRISPR, disease, cancer

## Abstract

With the development and wide usage of CRISPR technology, the presence of R-loop structures, which consist of an RNA–DNA hybrid and a displaced single-strand (ss) DNA, has become well accepted. R-loop structures have been implicated in a variety of circumstances and play critical roles in the metabolism of nucleic acid and relevant biological processes, including transcription, DNA repair, and telomere maintenance. Helicases are enzymes that use an ATP-driven motor force to unwind double-strand (ds) DNA, dsRNA, or RNA–DNA hybrids. Additionally, certain helicases have strand-annealing activity. Thus, helicases possess unique positions for R-loop biogenesis: they utilize their strand-annealing activity to promote the hybridization of RNA to DNA, leading to the formation of R-loops; conversely, they utilize their unwinding activity to separate RNA–DNA hybrids and resolve R-loops. Indeed, numerous helicases such as senataxin (SETX), Aquarius (AQR), WRN, BLM, RTEL1, PIF1, FANCM, ATRX (alpha-thalassemia/mental retardation, X-linked), CasDinG, and several DEAD/H-box proteins are reported to resolve R-loops; while other helicases, such as Cas3 and UPF1, are reported to stimulate R-loop formation. Moreover, helicases like DDX1, DDX17, and DHX9 have been identified in both R-loop formation and resolution. In this review, we will summarize the latest understandings regarding the roles of helicases in R-loop metabolism. Additionally, we will highlight challenges associated with drug discovery in the context of targeting these R-loop helicases.

R-loops are three-stranded nucleic acid structures consisting of an RNA–DNA hybrid and a displaced single-stranded (ss) DNA. They play critical roles in various biological processes, including transcriptional regulation and replication, genomic instability, class switch recombination in B cells, DNA damage and repair, and telomere maintenance (1, 2, 3, 4, 5). Helicases are a group of molecular motors that utilize the energy from nucleoside triphosphate hydrolysis to unwind and remodel DNA and RNA molecules, or protein–nucleic acid interactions (6, 7, 8, 9). Notably, some helicases also possess strand annealing activity (10). This unique property allows them to play crucial roles in R-loop biogenesis. They can utilize their unwinding activity to separate double-stranded (ds) DNA, facilitate RNA invasion, or use their strand annealing activity to promote RNA to hybridize to ssDNA, enabling R-loop formation. Conversely, helicases can also utilize their unwinding activity to separate RNA–DNA hybrids to resolve R-loops. Indeed, various helicases have been implicated in R-loop assembly and disassembly. For example, SETX (Senataxin), AQR (Aquarius), WRN (Werner syndrome), BLM (Bloom syndrome), RTEL1 (Regulator of telomere elongation helicase 1), PIF1 (Petite integration factor 1), FANCM (Fanconi anemia complementation group M), ATRX (alpha-thalassemia/mental retardation, X-linked), CasDinG (CRISPR-associated DinG protein), and several DEAD/H-box proteins are reported to resolve R-loops. In contrast, helicases such as Cas3 and UPF1 (Up-frameshift protein 1) are reported to stimulate R-loop formation. Moreover, helicases such as DDX1, DDX17, and DHX9 are involved in both R-loop formation and resolution.

Several excellent reviews are available for R-loops and their biological functions (1, 2, 3, 4, 5). Specific enzymes in R-loop metabolism, including nuclease RNase H (11, 12, 13) and topoisomerase (14, 15), have been discussed extensively. However, a comprehensive understanding of helicases in R-loop biogenesis is missing. In this review, we will summarize the latest knowledge of the roles of helicases in R-loop metabolism. In addition, this review will address the challenges related to drug discovery efforts targeting helicases and R-loops.

## R-loop

**Figure 1:**
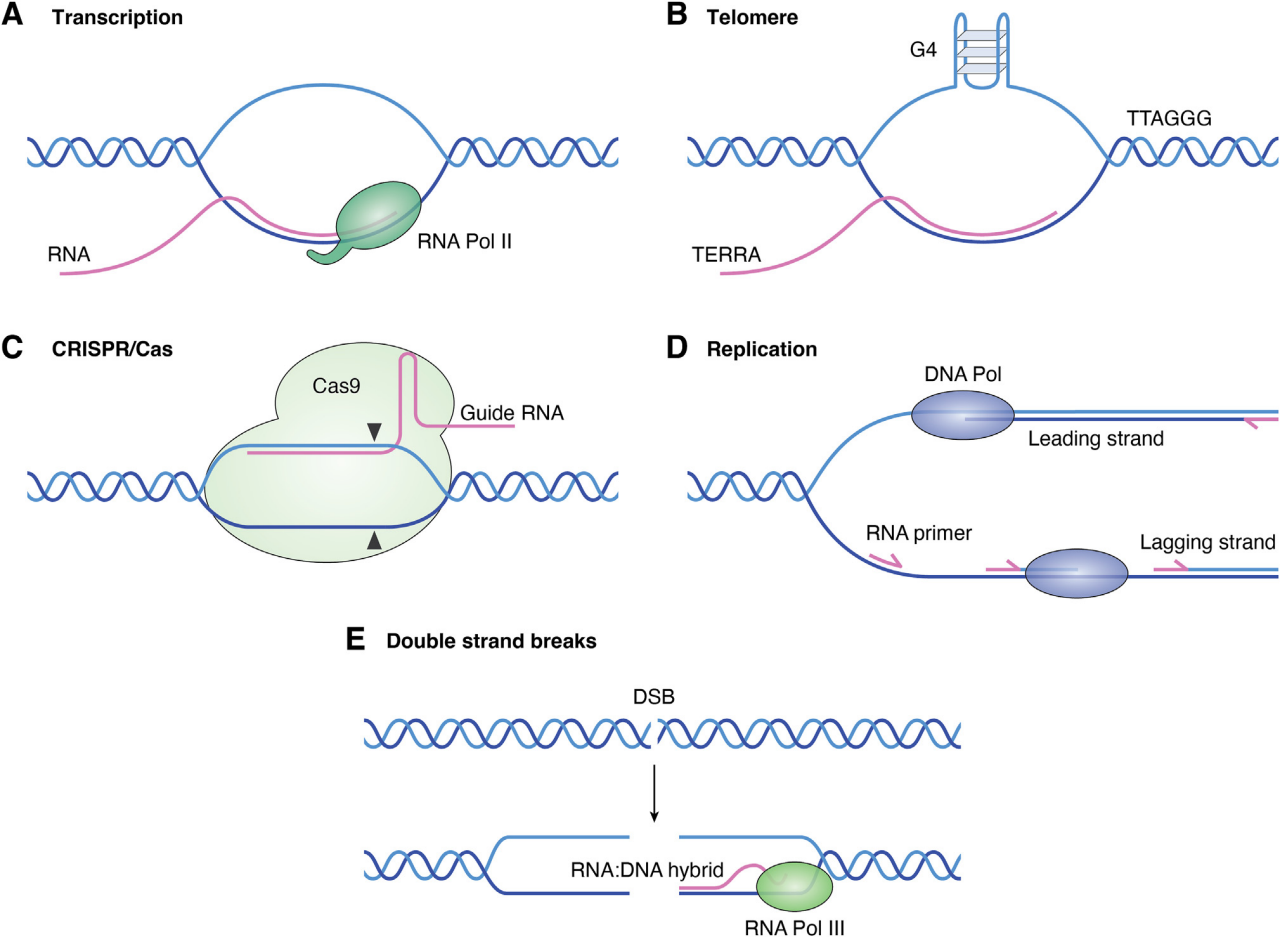
**Potential R-loops and RNA**–**DNA hybrids in cells.***A*, an R-loop forms during transcription, where a nascent RNA synthesized by RNA polymerase II can base pairs with a DNA template, forming an RNA–DNA hybrid and displaced non-template ssDNA. *B*, an R-loop forms at the telomere. Telomeres are transcribed into TERRA that binds to telomeric DNA, forming R-loop structures, leaving a displaced G-rich DNA strand that forms G4. *C*, an R-loop forms in CRISPR/Cas9. A guide RNA binds the target DNA strand while the nontarget strand is displaced, forming an R-loop structure, and Cas9 cleaves both strands. *D*, an RNA–DNA hybrid forms during DNA replication, where a primer RNA in a leading/lagging strand pairs with a DNA template. *E*, an RNA–DNA hybrid forms during DNA double-strand breaks (DSBs). A new RNA synthesized by RNA polymerase III at DSB pairs with a DNA template.

While R-loops were first observed *in vitro* in 1976 (16), the existence of an R-loop *in vivo* was not reported until 1995 in bacteria (17). R-loops were initially considered a by-product of transcription where the nascent RNA transcribed by RNA polymerase remains base paired with its template DNA, leaving the non-template ssDNA. Now, it is known that R-loops occur genome-wide and are present in all organisms, from bacteria to humans (18, 19, 20, 21). Indeed, the development and wide use of CRISPR techniques demonstrate that R-loop structures exist naturally in cells. In the context of the CRISPR system, an ssRNA (guide RNA) hybrids with a targeted ssDNA, displaces an ssDNA, and forms an R-loop structure; an endonuclease Cas protein then cleaves the targeted DNA (5, 22). R-loops and RNA–DNA hybrids can also form during transcription, DNA replication, double-strand breaks (DSBs) repair, and at telomeres (Fig. 1).

Generally, R-loops are categorized as either physiological or pathological (1, 5). The physiological R-loops are programmed, whereas the pathological R-loops are nonprogrammed. Pathological R-loops can threaten genomic stability in various ways, such as generating transcription-replication collisions, single-stranded DNA breaks (SSBs), and DSBs (23, 24). On the other hand, the physiological functions of R-loops comprise immunoglobulin class switching of B cells in vertebrates (25), gene editing using CRISPR-Cas9 (26), mitochondrial DNA replication (27), specific regulatory steps in transcription (28), DSB repair (29), CGG repeat contraction (30), and maintaining telomere homeostasis (31). Furthermore, RNA polymerase III can catalyze transcription at DSBs, forming a transient RNA–DNA hybrid to protect the 3ʹ overhang from degradation before replication protein A (RPA) binding, demonstrating the RNA–DNA hybrid at DSB is an essential repair intermediate in the process of homologous recombination (HR)-mediated DSB repair (32). These findings indicate R-loops can both cause DSB and facilitate DSB repair, leading to negative or positive outcomes depending on the molecular environment of the R-loop. Thus, R-loop homeostasis must be tightly regulated to balance its physiological and pathological roles properly.

Several neurodegenerative disorders and various cancers are associated with dysregulated R-loops. For example, more than half of patients with the neuroinflammatory Aicardi-Goutières syndrome (AGS) have biallelic mutations in RNase H2 (33, 34). R-loop is also implicated in ataxia with oculomotor apraxia type 2 (AOA2) (35) and juvenile amyotrophic lateral sclerosis (ALS4) (36). AOA2 is an autosomal recessive disease associated with SETX loss of function, while ALS4 is an autosomal dominant disease provoked by toxic gain-of-function mutations in SETX (37). In fragile X syndrome, the expansion of CGG trinucleotide repeats in the *FMR1* gene leads to R–loop formation that contributes to DNA damage and chromosomal instability (38). Moreover, mutations in the RNA binding protein TDP-43 can cause aberrant R-loop formation, damaging DNA and contributing to ALS (39, 40). In the context of tumorigenesis, mutations in DDX41 are associated with myeloid neoplasms myelodysplastic syndromes (MDS) and acute myeloid leukemia (AML) (41), and excessive R-loops are found in DDX41 mutated cells (42, 43). Similarly, EWS-FIL1 fusion in Ewing sarcoma also induces R-loop accumulation and perturbs HR repair, potentially mediating chemosensitivity (44).

## R-loop binding proteins

Several groups have employed affinity pull-down and BioID (proximity-dependent biotin identification) techniques to identify R-loop binding proteins (45, 46, 47, 48, 49, 50) (Table S1). Kumar *et al.* (51) analyzed the five datasets and found only 12 common R-loop binding proteins: DDX5, NAT10, NPM1, NOP2, DDX18, NOP58, ALYREF, U2AF1, ILF3, RBM14, PDCD11, and MYBBP1A. More recently, Marchena-Cruz *et al.* used siRNA screening coupled with AID-induced DSBs as a readout and identified 46 proteins that affect R-loop homeostasis (52) (Table S1). These seven datasets revealed varying results, with no single protein present in all datasets. Using similar approaches, such as affinity pull-down with S9.6 antibody by Cristini *et al.* (45) and Wu *et al.* (46), affinity pull-down with Myc-tagged RNase H (50), or BioID with RNase H as a bait by Mosler *et al.* (48) and Yan *et al.* (49), their datasets are largely different (Table S1). Collectively, these results suggest R-loop binding proteins are highly variable depending on study conditions and thus additional investigations are required to fully elucidate the identity of R-loop binding proteins.

The diversity of identified R-loop binding proteins can be attributed to several factors. Firstly, different methods employed in these studies may lead to different R-loop binding proteins. For instance, affinity pull-down can capture stable protein complexes but not proteins with weak or transient interactions. While BioID can capture weak or transient binding partners, the increased sensitivity may also detect potential indirect interactions with R-loops. Secondly, flaws in different methods may yield false positive—non-specific binding proteins. Indeed, the S9.6 antibody has a non-specific binding with dsRNA (53, 54), RNase H consists of two enzymes (H1 and H2) with specificities in the cell cycle and subcellular location (55, 56), and AID-based screening may detect ssDNA other than R-loops (52). Thirdly, the different cell types used in these studies such as HeLa, HEK293, U2OS, human B cells, and mouse embryonic stem cells may contribute to variable results. Finally, there may be a dynamic change of R-loops in cells and thus the associated R-loop binding proteins may also undergo dynamic changes, resulting in increased diversity amongst different datasets. Nevertheless, despite the different observations in R-loop binding proteins, helicases are consistently identified in all studies (Table S1, highlighted in yellow).

## R-loop resolution helicases

RNase H1 and H2 are nucleases that specifically target and degrade the RNA molecule within RNA–DNA hybrid of R-loops (57). RNase H1 is primarily localized in the mitochondria (55), whereas RNase H2 displays G2/M-phase specific expression (56). We believe a large number of nucleases that target and degrade ssDNA or RNA released from RNA–DNA hybrids by helicases would be more robust in eliminating R-loops. Consistent with the initial observation that R-loops are a threat to genome stability, many helicases have been reported to prevent R-loop accumulation, including SETX, AQR, WRN, BLM, FANCM, SMARCAL, PIF1, DDX5, ATRX, and CasDinG (Table 1).

**Table 1:** Helicases involved in R-loop metabolism

Helicase family	Name		Role in R-loop		Disease or main phenotype/biological function	Note	References
			Form R-loop	Resolve R-loop			
	Human	Yeast					
SF1	SETX	Sen1		√	AOA2 and ALS4		(58, 149)
	AQR			√	Splicing factor	Aka IBP160	(150)
	UPF1	Upf1	√		Nonsense mRNA degradation surveillance		(31)
	PIF1	Rrm3, ScPif1, SpPfh1		√	Genomic instability in the nucleus and mitochondria	*S. cerevisiae* has two members: Rrm3 and ScPif1; *S. pombe* has one: Pfh1	(151)
SF2	WRN			√	Werner syndrome		(63)
	BLM	Sgs1		√	Bloom syndrome		(65)
	RTEL1			√	Telomere shortening	Interacts with Poldip3	(68)
	FANCM	Mph1		√	Congenital abnormalities, pancytopenia, infertility, and cancer proneness		(70, 71, 152)
	Polymerase θ (POLQ)			√	Xeroderma Pigmentosum	Helicase domain (1–894 aa) unwinds RNA–DNA hybrids *in vitro*	(73)
	CasDinG (Bacteria)			√			(74, 75)
SNF2	ATRX	Rad54		√	ATRX syndrome	A recent report shows ATRX is unable to resolve R-loops (49)	(72)
	SMARCAL1			√	Schimke Immunoosseous Dysplasia	Aka HARP, ATP-driven annealing helicase	(71, 153)
	ZRANB3			√	African-specific type 2 diabetes	Aka annealing helicase 2 (AH2)	(71)
DEAD-box	DDX1		√	√	Retinoblastoma and neuroblastoma		(100, 103)
	DDX5	Dpb2		√			(37, 76, 77, 78, 79, 154, 155)
	DDX17		√	√		Aka p68 and p72, contains strand annealing activity	(79, 105, 109)
	DDX18			√		PARP-1 mediated	(80)
	DDX19			√			(81)
	DDX21			√		Coordinates with SIRT7 or JMJD3	(82, 83)
	DDX23			√			(84)
	DDX41			√			(42, 48)
	DDX47			√			(52)
DEXD-box	DDX39B			√	Autoimmune disease	Aka UAP56	(85)
DEXH-box	MTR4			√	Trichohepatoenteric Syndrome		(61)
	DHX9		√	√		Aka RNA helicase A (RHA)	(45, 119)
	Cas3 (Bacteria and archaea)		√	√		Cas3 resolves R-loop by its nuclease activity	(89, 92)

**Figure 2:**
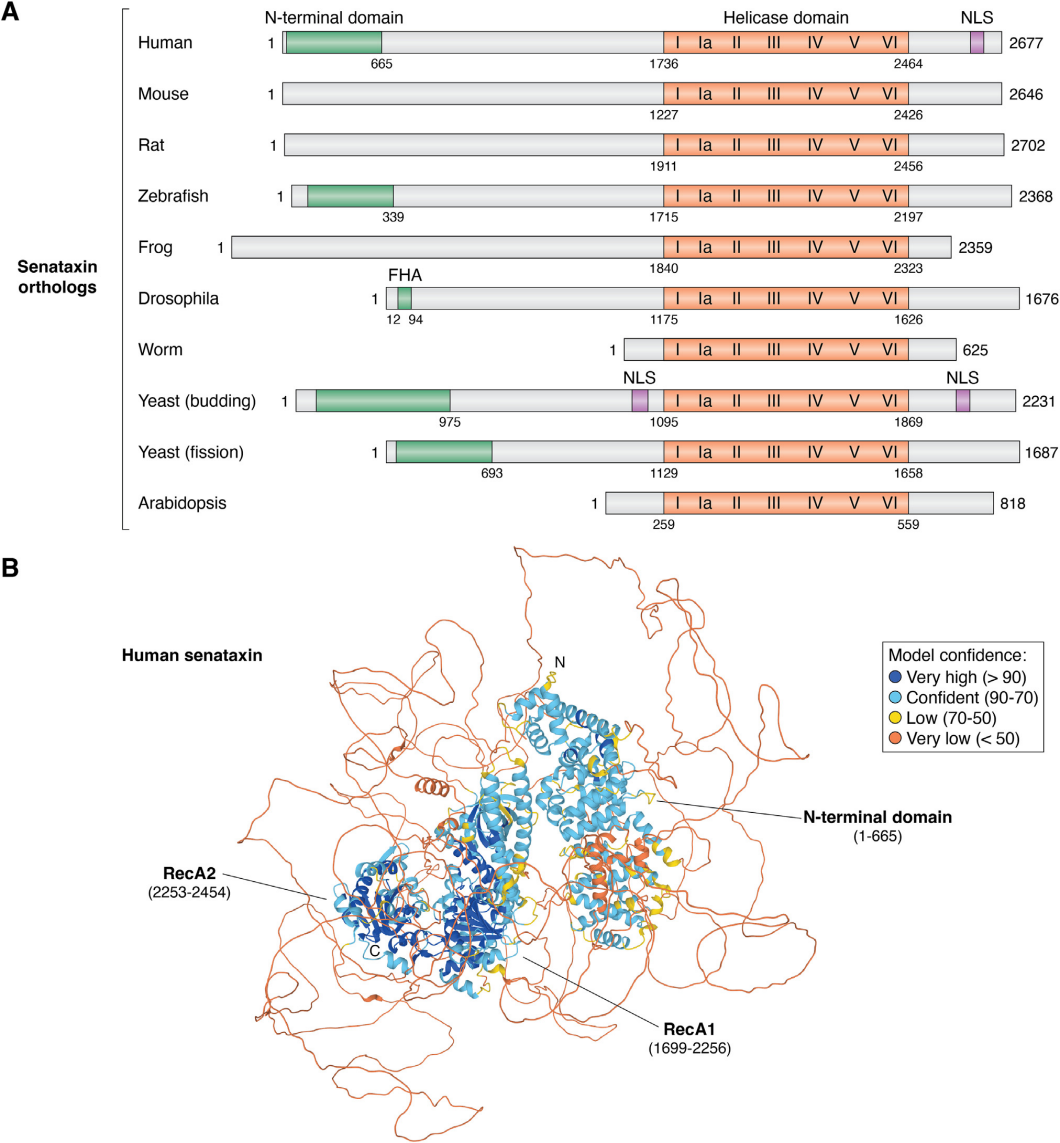
**Alignment of senataxin orthologs and predicted human senataxin structure.***A*, the conserved helicase core domain is indicated in orange, and the seven helicase motifs and the accessory domains are indicated. The number of amino acids in each helicase is shown on the *right*. The exact boundaries of domains may differ. Most sequences are from Uniprot: human (Q7Z333), mouse (A2AKX3), rat (A0A8I5ZYQ6), *Drosophila* (B7Z0D7), zebrafish (A0A8N7UR12), baker’s yeast (Q00416), Arabidopsis (B6SFA4), and CasDinG (Q2FGY5); some are from databases: Wormbase (Eri-7, CE36108), Pombase (SPAC6G9.10c), and the frog is from Ref (156). *B*, predicted human senataxin structure by the Alphafold2. The conserved RecA1 and RecA2 domains, the structured N-terminal domain, the first (N) and last residue (C) are indicated. Amino acids are colored based on their per-residue confidence score (pLDDT), which values can be between 0 and 100. Low values indicate low confidence and high numbers indicate very confident predictions. Amino acids are either colored *orange* (pDLLT <50), *yellow* (70> pDLLT >50), *light blue* (50< pDLLT >70), or *dark blue* (pDLLT >90). Regions with pLDDT <50 may be unstructured in isolation. FHA, forkhead associated domain; NLS, nuclear localization signal.

Yeast protein Sen1 and its human ortholog SETX are the first and foremost well-characterized R-loop resolving helicases. In 2011, Mischo *et al.* (58) found that loss of Sen1 results in R-loop accumulation and DNA damage in yeast, and Skourti-Stathaki *et al.* (59) found that human SETX protein resolves RNA–DNA hybrids. As a member of the SF1B class of helicases, Sen1 and SETX unwind R-loops and RNA–DNA duplexes with a 5′ to 3′ polarity (60). Additional mouse model studies showed Mtr4 (an exosome RNA helicase) and Setx work collaboratively in unwinding RNA exosome-sensitive noncoding RNA (ncRNA) from RNA–DNA hybrids (61). This cooperative action between Mtr4 and Setx is involved in the processing and resolution of R-loops. SETX is conserved across species, with a helicase domain at its C-terminus (Fig. 2*A*). Besides its structured N-terminal domain and conserved helicase RecA1 and RecA2 domains, a large portion of the human SETX protein is unstructured (Fig. 2*B*). It remains to be determined whether the disordered regions are involved in protein–protein interaction and the cleft between the N-terminal domain and RecA1 domain is involved in R-loop binding. So far, much biochemical work has been done using its helicase domain, and their full-length protein has yet to be studied.

In addition to Sen1/SETX, many helicases have been reported to prevent R-loop accumulation. AQR plays a critical role in the HR repair pathway by promoting the loading of RPA and Rad51 onto ssDNA regions and resolving R-loops that can interfere with DNA repair (62). R-loops are accumulated in Werner syndrome cells (63). WRN and WRNIP1 (WRN-interacting protein 1) are required to counteract R-loop accumulation (64). BLM, another RECQ family helicase, has also been reported in resolving R-loops (65, 66, 67). R-loop accumulation has been observed in cells lacking RTEL1, which is known to localize at telomeres and can resolve TERRA-containing R-loops in these regions (68, 69). R-loop accumulates in FANCM-depleted cells and FANCM protein can unwind RNA–DNA hybrids *in vitro via* its translocase activity (70). A related study also suggests three helicases, FANCM, SMARCAL1, and ZRANB3, are important for R-loop removal and genome stability in human cells (71). ATRX suppresses R-loops in transcribed telomeric repeats (72). POLQ possesses proficient unwinding abilities for RNA–DNA hybrids *via* its helicase domain (residues 1–894) and demonstrates a preference for unwinding the lagging strand during replication fork progression (73). CasDinG (CRISPR-associated DinG) helicase is reported to resolve R-loops (74, 75), as discussed in the next section along with Cas3.

**Figure 3:**
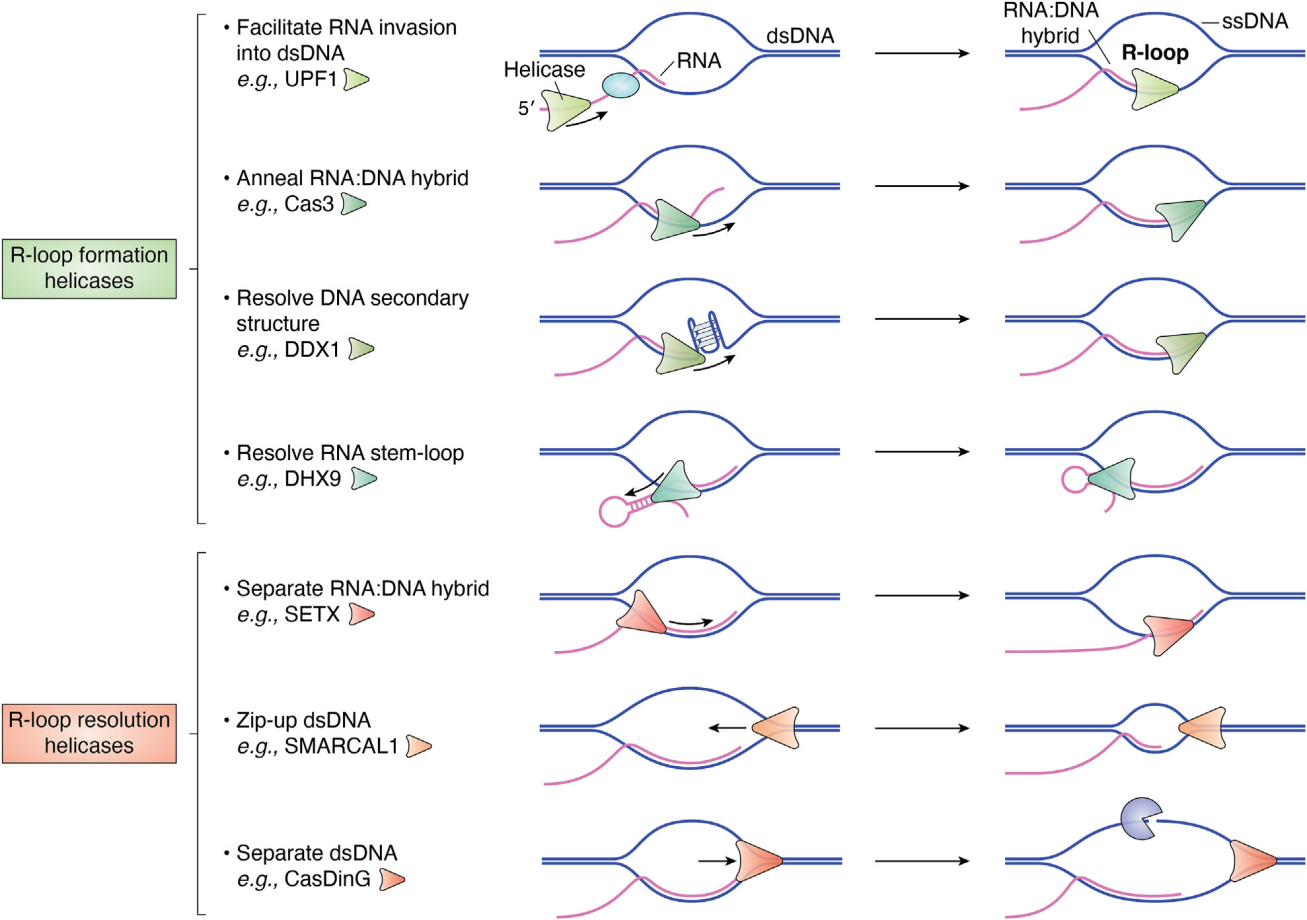
**Potential roles of helicases in R-loop metabolism.** For R-loop formation helicases (*greentriangle*), they may utilize their helicase/translocase activity to remove any secondary structures or R loop suppressing proteins (*blue oval*) on the RNA to facilitate the invasion of RNA into dsDNA (*e.g.*, UPF1), their strand annealing activity to facilitate RNA–DNA hybrid formation (*e.g.*, Cas3) or their unwinding activity to unwind secondary structures on ssDNA(*e.g.*, DDX1) or ssRNA (*e.g.*, DHX9) and facilitate RNA–DNA hybrid formation. For R-loop resolution helicases (*redtriangle*), they may utilize their unwinding activity to separate RNA–DNA hybrids (*e.g.*, SETX), utilize their strand annealing activity to anneal dsDNA and push away ssRNA (*e.g.*, SMARCAL1), or separate dsDNA and lead to long ssDNAs that are degraded by nucleases (*gray* three-quarter *circle*) (*e.g.*, CasDinG).

Several helicases belonging to the DEAD/H-box family have been reported to prevent R-loop accumulation. DDX5 has been reported to resolve R-loops by different groups (37, 76, 77, 78, 79). DDX18 can inhibit the accumulation of R-loops mediated by PARP-1 (poly[ADP-ribose] polymerase-1). Specifically, PARP-1 generates PAR polymers serve as a scaffold that facilitates the binding between DDX18 and R-loops, and its activation results in reduced R-loops (80). DDX19 enters the nucleus in an ATR-dependent manner following DNA damage to remove R-loops (81). DDX21 also unwinds RNA–DNA hybrids and cooperates with enzyme sirtuin seven to suppress R-loops at specific genes (82, 83). The phosphorylation of DDX23 is essential for protection against the accumulation of R-loops, and this relies on its RNA helicase activity (84). *In vitro* experiments demonstrated that DDX41 can effectively resolve RNA–DNA hybrids by its unwinding activity, and the depletion of DDX41 leads to the accumulation of R-loops (42, 48). DDX47’s ability to unwind RNA–DNA hybrids contributes to maintain low levels of harmful R-loops (52). DDX39B has been shown to unwind RNA–DNA hybrids *in vitro* and suppress co-transcriptional R-loops across the genome (85) (Fig. 3).

## R-loop formation helicases

In contrast to resolving R-loop structures, several helicases have been reported to facilitate R-loop formation (Table 1). These include prokaryotic Cas3 and human helicase UPF1.

Cas3 is a signature protein of the type I CRISPR-Cas systems and it is an ATP-dependent helicase (86). Belonging to the SF2 family and the DExD/H-box family, Cas3 contains a helicase domain in its C-terminus and a nuclease domain in its N-terminus. Although the Cas3 protein separates duplex DNA in a 3′ to 5′ direction *in vitro* (86, 87), it appears that Cas3 is not directly involved in DNA bubble creation in the initial steps of R-loop formation (88). During type I CRISPR immunity in prokaryotes, guide RNA (gRNA) with Cas3 and other cascade proteins forms a Cascade complex that promotes strand separation of the duplex DNA. Subsequently, gRNA recognizes and hybridizes with the complementary protospacer DNA, which results in the formation of an R-loop. Because Cas3 can anneal RNA–DNA hybrids (89), it induces the formation of RNA–DNA hybrids by hydrogen-bonded base pairs between complementary RNA (gRNA) and targeted DNA. Further studies show that Cas3 is activated at the Cascade-marked R-loop region (90, 91) and preferentially cleaves the nontarget-strand DNA ∼12 nt into the R-loop region. Cas3 then moves 3′ to 5′ direction driven by ATP hydrolysis and catalyzes a similar degradation action on the target strand using its nuclease activity. However, it remains unknown whether Cas3 unwinds RNA–DNA hybrid to facilitate R-loop disassembly (92). Another bacterial helicase is DinG (damage-inducible gene G), which belongs to SF2 and iron-sulfur (Fe-S) cluster family (93, 94). CasDinG, the hallmark of the type IV CRISPR system, is an ssDNA-stimulated ATP-dependent 5′-3′ DNA and RNA–DNA helicase (74, 75). It contains two RecA-like domains and three accessory domains (N-terminal, arch, and Fe-S) (74). Recent evidence suggests that CasDinG is not involved in R-loop formation but rather in R-loop resolution (75) (Fig. 3). Unlike Cas3 and CasDinG, Cas9, the widely used type II CRISPR system, contains two nuclease domains and does not have energy-dependent helicase activity. It seems that no helicase is involved in Cas9/R-loop complex formation or resolution (95).

UPF1 is an SF1 helicase with high processive and translocase activities (96). Human UPF1 has two isoforms: a short isoform containing 1118 amino acid residues, and a long isoform with an additional 11 amino acids in its regulatory loop (97). The short isoform is the most studied and accounts for approximately 80% of UPF1 mRNA in humans. UPF1 is best known for its role in nonsense-mediated mRNA decay (NMD) (98). Using a siRNA screening, Ngo *et al.* (31) identified UPF1 as an essential factor promoting the formation of R-loops that can stimulate DNA resection in DSB repair. How UPF1 generates R-loops remains unclear. One hypothesis is that UPF1 can bind at the 5′ end of RNA transcripts near DSBs and while translocating toward the 3′ end of RNA, it can remove RNA secondary structures or proteins that suppress R-loops. This process facilitates the annealing of these RNAs to ssDNA or dsDNA (Fig. 3). Liu *et al.* (32) reported that RNA polymerase III catalyzes transcription at DSBs, forming a transient RNA–DNA hybrid to protect the 3′overhang from degradation before RPA binding. Whether UPF1 and RNA pol III cooperate at DSBs for R-loop formation remains unknown. However, Ngo *et al.* (31) suggested that R-loops formed at DBSs facilitate DNA resection, while Liu *et al.* (32) proposed that R-loops formed at DBSs prevent DNA resection. Further investigation is required to clarify the differential roles of R-loops in DNA end resections.

While it is likely that new R-loop formation helicases will be identified in future studies, it is equally important to better understand the underlying mechanism of how R-loops form due to helicase activity. Specifically, it is unclear whether the stimulation of R-loop formation is due to a helicase’s unwinding activity that separates dsDNA and facilitates the invasion of RNA into duplex DNA, or due to the helicase’s strand annealing activity that stimulates the binding of RNA to ssDNA, or due to other unknown co-factors.

## R-loop resolution and formation helicases

Interestingly, some helicases such as DDX1, DDX17, and DHX9 helicases have been reported to act in both R-loop formation and resolution (Table 1).

DDX1 is a helicase involved in various cellular processes including RNA metabolism (99), DNA repair (100, 101), and mitochondrial activity (102). Li *et al.* (101) showed that DDX1 co-localizes with γH2AX and phosphorylates ATM at sites of DSBs and that it has ADP-dependent RNA–DNA unwinding activities. Subsequently, the same lab found that DDX1 can remove RNA from RNA–DNA hybrids and facilitate HR at the DSBs sites (100). In contrast, Ribeiro de Almeida *et al.* (103) reported that DDX1 converts RNA G-quadruplex structures into R-loops to promote IgH class switch recombination (CSR). This finding suggests that DDX1 directly binds G4 structures present within intronic switch RNA, enabling the targeting of activation-induced cytidine deaminase (AID) to S-region DNA, thus facilitating CSR. Despite the different consequences observed regarding the R-loop, in both cases DDX1 exploits its unwinding activity to resolve the R-loop at DSB by removing the RNA strand from the RNA–DNA hybrid (100), and DDX1 uses its unwinding activity to resolve G4 RNA structures and smooth RNA strands to facilitate the formation of RNA–DNA hybrid at the AID switch region (103) (Fig. 3).

DDX17 has RNA unwinding (104), strand annealing, and branch migration activities (105), and functions in microRNA biogenesis (106, 107), transcription (107), and splicing (104, 107). Previous studies indicated DDX17 is involved in DNA damage repair (108). However, the molecular mechanism is unknown. Bader *et al.* (109) found that DDX17 facilitates R-loops formation at DSBs. Interestingly, this role is prominent in the genome regions naturally deficient for RNA–DNA hybrids. In contrast, Polenkowski *et al.* (79) studied the roles of DDX17 and DDX5 in R-loops and observed that DDX5- and DDX17-depleted cells have accumulated R-loops. Furthermore, they observed that DDX17 and DDX5 proteins can unwind the RNA–DNA hybrid substrates. Moreover, overexpression of DDX5 or DDX17 suppressed the R-loop accumulation in cells. Yet, how DDX17 promotes R-loop formation at DSB sites (109) and prevents R-loop accumulation in transcription elongation (79) remains unclear and need additional investigation. Overall, these findings suggest DDX17’s function in R-loop regulation appears to be substrate and context-specific.

DHX9, also known as RHA (RNA helicase A), participates in RNA processing (110), transcription (111, 112), and replication (113). Monoallelic variations in DHX9 cause neurodevelopment disorders and Charcot-Marie-Tooth disease (114). Using affinity purification with the S9.6 antibody and mass spectrometry, Cristini *et al.* (45) found that DHX9 is one of the top R-loop binding proteins. Furthermore, they discovered that the depletion of DHX9 triggers R-loop and γH2AX accumulation, indicating that DHX9 resolves R-loops and prevents DSBs. Several other groups also reported the R-loop resolving function of DHX9 (115, 116, 117). Indeed, DHX9 protein can unwind RNA–DNA hybrids *in vitro* (118). However, Chakraborty *et al.* (119) found that DHX9 promotes R-loop formation in U2OS cells. They proposed that DHX9 may promote R-loop formation by unwinding secondary structures in the nascent RNA strand, generating a free RNA end that can invade duplex DNA to form an RNA–DNA hybrid (Fig. 3). The mechanisms underlying the opposing functions of DHX9 on R-loop are not clear. One possibility is that the dual role of DHX9 may be regulated by its interaction with various partners, such as C1orf109L (120), USP42 (115), ATAD5 (121), RNF168 (116), TDRD3 (117), ADAR1 (122), and NUSAP1 (123).

Interestingly, complete knockout of Ddx1 (124), Ddx17 (125), or Dhx9 (126) is embryonic lethal in mouse models, suggesting their importance in development. Whether dysregulated R-loops are present in these animals and how they contribute to the observed phenotype remains to be investigated.

## Questions remaining for R-loop helicases

Despite the significant progress made during the past three decades in understanding the biogenesis and functions of the R-loop, many questions still need to be answered, primarily related to the roles of helicases in the homeostasis of R-loops.

### How do helicases recognize and bind R-loop structures?

**Figure 4:**
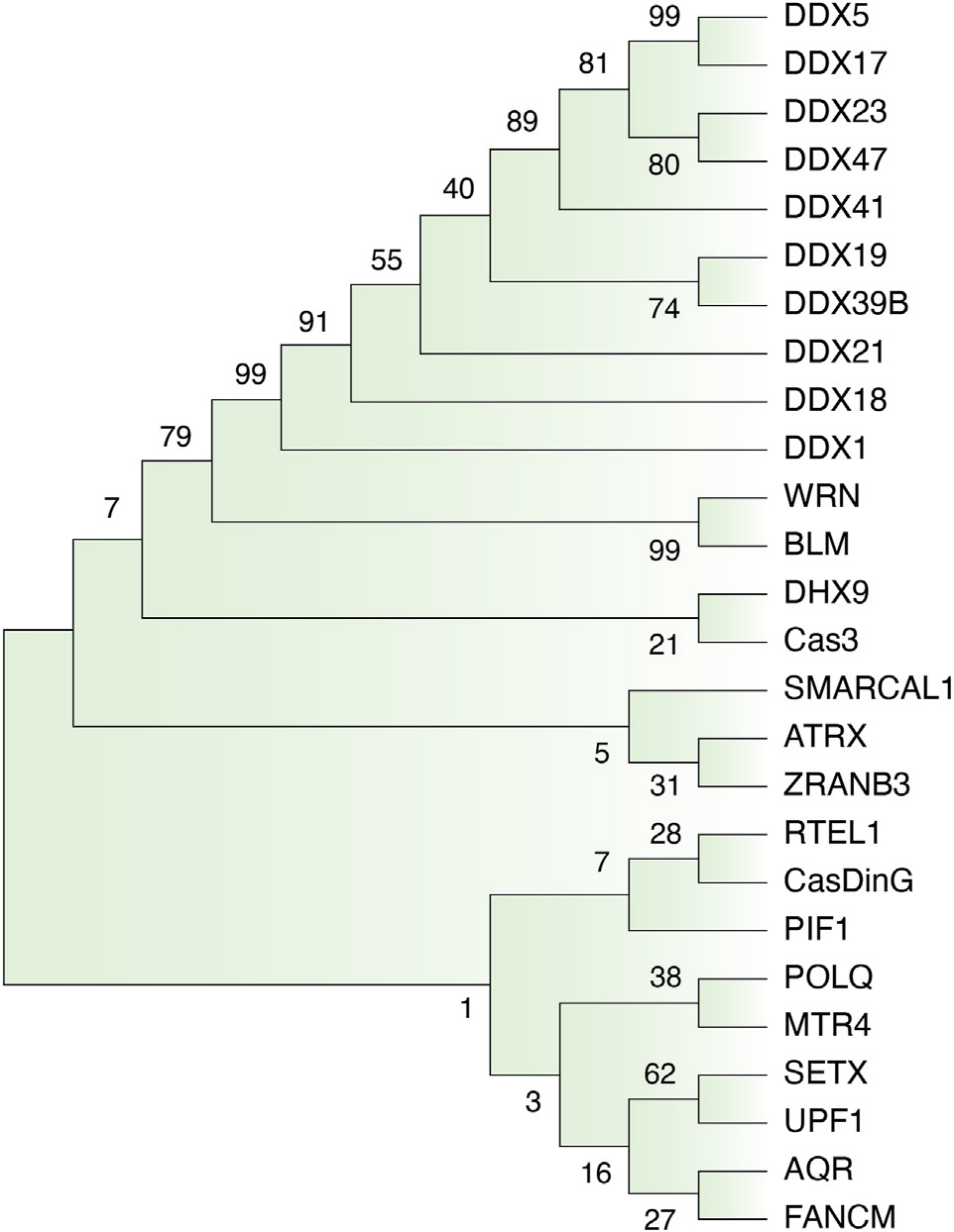
**Phylogenetic analysis of R-loop helicases.** The phylogenetic tree was constructed by the neighbor-joining method using the MEGA11 software with 1000 bootstrap replicates. The optimal tree is shown. Bootstrap values are shown at nodes. This analysis involved 26 amino acid sequences, 24 are from *Homo sapiens*, Cas3 from *Streptococcus thermophilus*, and CasDinG from *Pseudomonas aeruginosa* (Table 1).

There are writers, readers, and erasers for epigenetic modifications such as DNA methylation and histone modifications. While nucleic acids predominantly exist as dsDNA and ssRNA, RNA–DNA hybrids and related R-loops may exist transiently like epigenetic modifications. If helicases are considered significant writers and erasers, could they also be readers for R-loops? Helicases can recognize and bind to R-loops through different potential mechanisms: (1) RNA–DNA hybrid recognition: Many helicases have a specific affinity for RNA–DNA hybrids and the RNA component of the R-loop. For example, the helicase domain of the SETX exhibits high affinity towards hybrids with a 5′ or 3′ ssRNA overhang and no binding with dsDNA (60). (2) 3D structure recognition: For instance, FMRP (Fragile X mental retardation protein) binds R-loops, including ssDNA, dsDNA, RNA, and RNA–DNA hybrids, through its N-terminal folded core and C-terminal intrinsically disordered region (127). Whether the large and disordered regions in SETX (Fig. 2*B*) are involved in R-loop binding remains to be determined. (3) Protein–protein interactions: Sometimes, helicases may not recognize R-loop structures directly. Thus, other R-loop binding proteins are required to recruit the helicase to the R-loop site. Indeed, DDX18 was discovered to control the balance of R-loop metabolism, and this process is mediated by the activity of PARP-1, whose product PAR polymers enhance the recruitment of DDX18 proteins to R-loops (80). Using MEGA11 software (128), we performed phylogenetic analysis and found that SF2, especially DEAD-box helicases, are grouped; however, no clear clade is classified among SF1 and SNF1 helicases evidenced by low bootstrap valves (Fig. 4). Close relationship is observed between DDX5 and DDX17, WRN and BLM, DDX23 and DDX47, DDX19 and DDX39B, SETX and UPF1, indicating the property of R-loop metabolism is conserved among these helicases but not across helicase families. Structures of R-loops formed in CRISPR-Cas complexes have provided us specific snapshots of R-loop formation by Cas proteins (75, 92, 129, 130). However, more structural studies are needed to fully determine the role of these helicases in R-loops metabolism (Table 1). For example, whether any conserved motifs or domains evolved among R-loop helicases, do helicases only recognize and bind RNA–DNA hybrids or do they also recognize the displaced ssDNA or adjacent dsDNA? Importantly, different helicases can have distinct mechanisms for recognizing and binding R-loops, and our current understanding of these mechanisms remains incomplete. Determining the recognition signals and the involvement of binding sites will provide insight into the initial steps of R-loop processing.

### How do helicases execute their roles in R-loop metabolism?

The involvement of multiple helicases in R-loop biogenesis raises questions about their specific functions and interactions. Why numerous helicases are involved in R-loop metabolism and whether they cooperate, function redundantly, or act independently are all active areas of research. Marchena-Cruz *et al.* (52) showed that DDX47 has a complementary function with SETX, a partially redundant role with DDX23, and an independent function with DDX39B in R-loop metabolism. Conversely, some helicases like DDX1, DDX17, and DHX9 play dual roles in R-loop biogenesis. The mechanisms that regulate these helicases in both R-loop formation and R-loop resolution remains poorly understood. Indeed, while the annealing helicase SMARCAL1 (aka HARP) was reported to anneal dsDNA, pushing out the RNA strand in the R-loop (71), why SMARCAL1 does not anneal RNA–DNA hybrids to promote R-loop formation needs investigation. Like many other enzymes, R-loop helicases may have context-dependent functions, such as those concerning tissues, developmental stages, cell types, cell cycles, specific loci, and specific substrates, which need further investigation.

### How do helicases coordinate with other R-loop binding/processing proteins?

Besides helicases, various proteins such as nucleases, topoisomerases, RNA polymerases, ssDNA binding proteins, and RNA binding proteins (Table S1) work in concert to preserve biologically important R-loops while ensuring harmful R-loops are resolved quickly. For example, yeast RAD27 and human FEN1, a flap endonuclease, cleave the RNA of R-loop structures (131, 132). Additionally, endonuclease Sae2/CtIP prevents R-loop accumulation in eukaryotic cells (133). Single-stranded DNA-binding proteins such as RPA also co-localize with RNase H1 and facilitate R-loop removal (134, 135, 136). Furthermore, many helicases complex with other proteins to function in R-loop metabolisms, such as the DDX5/17-THOC5/6-CDK12 complex (79), DDX5-TOP3B complex (77), DHX9-TDRD3 complex (117), RTEL1-Poldip3 complex (68), and FANCM-SMARCAL1-ZRANB3 complex (71). The mechanisms of how helicases coordinate with these proteins to regulate R-loops' homeostasis remain unclear.

### What is the role of epigenetic factors in R-loop metabolism?

Epigenetic factors, such as DNA methylation, histone modifications, and chromatin structure, play critical roles in molecular metabolism, and R-loop is no exception. The relationship between epigenetics and the R-loop is reciprocal: epigenetic factors regulate the assembly and disassembly of R-loops, while R-loops affect epigenetic modifications. Indeed, R-loops are associated with specific epigenomic signatures at promoters and terminators, including particular histone marks, chromatin binding factors, and DNA hypomethylation (137). It has been reported that the TET (ten-eleven translocation enzymes), which oxidize 5-methylcytosine (5mC) into 5-hydroxymethylcytosine (5hmC), promote R-loop formation by favoring the annealing of the nascent RNA to the template DNA strand (138). This observation suggests proteins involved in epigenetic regulation may also influence R-loop formation. Recently, Sun *et al.* (139) reported that increased H3K4me3 results in R-loop accumulation in *C. elegans*. Panatta *et al.* (140) found that p53-deficient cells have reduced S-adenosylmethionine (SAM), methyl donor, leading to reduced H3K9me3 that triggers R-loop accumulation. N6-methyladenosine (m6A) is one of the most common internal epigenetic changes occurred in RNA molecules, and it was found that knockout of BRCA1 expression leads to reduced m6A RNA methylation and increased R-loop (141). On the other hand, R-loop modulates epigenetics in coronary artery disease and non-small cell lung cancer (142). Despite these correlational observations, how epigenetic factors regulate the expression and biochemical activities of these helicases in the context of R-loops remains unclear. Thus, the relationship between R-loops and epigenetic factors requires further investigation.

## Conclusion

Over the past few decades, the study of R-loops has evolved from its discovery from initial negative to positive effects. Accumulating evidence suggests that R-loops play critical roles in transcriptional regulation and replication, genomic instability independent of replication stress, class switch recombination in B cells, and DNA damage and repair. Maintaining the balance of R-loops is critical in health and diseases. Notably, R-loop formation is involved in genetic mutations and genomic instability in cancer. Thus, it is essential to better study the mechanisms underlying the complexities of the R-loop interactome and to pinpoint the molecular network and dependencies across various cancer types. Global perturbations in transcription, replication, and RNA processing by oncogenes can also result in improper R-loop accumulation. The associated DNA damage is linked to mutations that contribute to cancer etiology. Future studies using advanced tools including bioinformatics, single-molecule imaging, CRISPR technology, and the Alphafold program will help us better understand the structure, function, interaction, and regulation of R-loops.

Targeting helicases with drugs is attractive but challenging. Helicases have multiple catalytic steps and enzymatic activities, including NTP binding, NTP hydrolysis, NDP release, DNA or RNA binding or release, unwinding, annealing, translocation, and ribonucleoprotein remodeling. They provide a rich source for targeting. Meanwhile, helicases participate in all DNA and RNA metabolism processes, including fundamental DNA replication, transcription, and translation. These properties make it difficult to selectively inhibit their role in one specific function, such as R-loop metabolism without affecting other processes. In addition, many R-loop helicases can function on different substrates, including dsDNA, dsRNA, G-quadruplexes, triplexes, Holliday junctions, and telomere structures. Thus, it remains to be determined whether a particular inhibitor can specifically inhibit a helicase’s activity on R-loop structures.

Nevertheless, attempts have been made to develop drugs targeting helicases such as WRN (143), BLM (144, 145), POLθ (146), DDX3 (147), and DDX41 (148). Additional work is needed to refine the functionalities of the existing compounds, to enhance their specificity, and to reduce off-target effects. Since many helicases interact with other proteins, blocking the protein-protein interaction is an attractive alternative to modulating R-loops. The discovery of compounds that interact with R-loops will enhance drug selectivity and facilitate the design of new effective combination therapies, making this process more exciting. Investigating helicase functions in the context of R-loop metabolism and translating future discoveries will accelerate the development of novel treatments against cancers.

## Supporting information

This article contains Supporting information (45, 46, 47, 48, 49, 50, 52).

## Conflict of interest

The authors declare that they have no conflicts of interest with the contents of this article.
